# Supplementary material for: Membrane characteristics tune activities of endosomal and autophagic human VPS34 complexes
Source: eLife. 2020 Jun 30;9:e58281. doi: 10.7554/eLife.58281 (PMC7326497; doi:10.7554/eLife.58281)
Supplement: Transparent reporting form [file elife-58281-transrepform.pdf]

eLIFE

Westbrook Centre, Milton Road
Cambridge CB4 1YG
UK

P 01223 855340
W elifesciences.org
T @elife

eLife's transparent reporting form

We encourage authors to provide detailed information *within their submission* to facilitate the interpretation and replication of experiments. Authors can upload supporting documentation to indicate the use of appropriate reporting guidelines for health-related research (see EQUATOR Network), life science research (see the BioSharing Information Resource), or the ARRIVE guidelines for reporting work involving animal research. Where applicable, authors should refer to any relevant reporting standards documents in this form.

If you have any questions, please consult our Journal Policies and/or contact us: editorial@elifesciences.org.

Sample-size estimation

- You should state whether an appropriate sample size was computed when the study was being designed
- You should state the statistical method of sample size computation and any required assumptions
- If no explicit power analysis was used, you should describe how you decided what sample (replicate) size (number) to use

Please outline where this information can be found within the submission (e.g., sections or figure legends), or explain why this information doesn't apply to your submission:

The GUV enzyme assays recorded an average of at least 15 GUVs for each time point. Preliminary experiments showed 15 measurements could distinguish activities of the most similar complexes. Initial rates were compared using analysis of covariance (ANCOVA). HDX measurements used three measurements for each of five time points, based on previous experience with membrane-binding complexes. A paired t-test was used to compare HDX observations with and without membranes.

Replicates

- You should report how often each experiment was performed
- You should include a definition of biological versus technical replication
- The data obtained should be provided and sufficient information should be provided to indicate the number of independent biological and/or technical replicates
- If you encountered any outliers, you should describe how these were handled
- Criteria for exclusion/inclusion of data should be clearly stated
- High-throughput sequence data should be uploaded before submission, with a private link for reviewers provided (these are available from both GEO and ArrayExpress)

Please outline where this information can be found within the submission (e.g., sections or figure legends), or explain why this information doesn't apply to your submission:

Each GUV assay was repeated three times, each on a different day, using the same lipid and protein preparations. Each time point is the average and standard deviation for at least 15 observations of GUVs. The figures show a representative assay. Peptides were included in the HDX assay, only if peaks for each of the three replicates for each time point met the quality thresholds described in the manuscript, as explained in the methods.

Statistical reporting

- Statistical analysis methods should be described and justified
- Raw data should be presented in figures whenever informative to do so (typically when N per group is less than 10)
- For each experiment, you should identify the statistical tests used, exact values of N, definitions of center, methods of multiple test correction, and dispersion and precision measures (e.g., mean, median, SD, SEM, confidence intervals; and, for the major substantive results, a measure of effect size (e.g., Pearson's r, Cohen's d)
- Report exact p-values wherever possible alongside the summary statistics and 95% confidence intervals. These should be reported for all key questions and not only when the p-value is less than 0.05.

Please outline where this information can be found within the submission (e.g., sections or figure legends), or explain why this information doesn't apply to your submission:

Paired, two-tail t-tests were used to compare each time point in the two states for HDX experiments. These t-tests are shown in the supplementary Files 4 and 5. Statistical tests to compare rates for GUV assays were a single-tail t-test. The threshold of confidence was chosen as 0.05.

Group allocation

- Indicate how samples were allocated into experimental groups (in the case of clinical studies, please specify allocation to treatment method); if randomization was used, please also state if restricted randomization was applied
- Indicate if masking was used during group allocation, data collection and/or data analysis

Please outline where this information can be found within the submission (e.g., sections or figure legends), or explain why this information doesn't apply to your submission:

Additional data files ("source data")

- We encourage you to upload relevant additional data files, such as numerical data that are represented as a graph in a figure, or as a summary table
- Where provided, these should be in the most useful format, and they can be uploaded as "Source data" files linked to a main figure or table
- Include model definition files including the full list of parameters used
- Include code used for data analysis (e.g., R, MatLab)
- Avoid stating that data files are "available upon request"

Please indicate the figures or tables for which source data files have been provided:

For the GUV assays, the time-courses are shown with error bars in Supplementary File 3. For HDX, all means and standard deviations of deuterium uptake are included in the supplementary excel files. (Supplementary Files 4 and 5).
